# Domain Specific Changes in Cognition at High Altitude and Its Correlation with Hyperhomocysteinemia

**DOI:** 10.1371/journal.pone.0101448

**Published:** 2014-07-02

**Authors:** Vijay K. Sharma, Saroj K. Das, Priyanka Dhar, Kalpana B. Hota, Bidhu B. Mahapatra, Vivek Vashishtha, Ashish Kumar, Sunil K. Hota, Tsering Norboo, Ravi B. Srivastava

**Affiliations:** 1 Defence Institute of High altitude Research, Defence Research and Development Organisation, C/o 56 APO, Leh-Ladakh, India; 2 Department of Statistics, Population Council, New Delhi, India; 3 Ladakh Institute of Prevention, Dambuchan, Leh-Ladakh, India; University Of São Paulo, Brazil

## Abstract

Though acute exposure to hypobaric hypoxia is reported to impair cognitive performance, the effects of prolonged exposure on different cognitive domains have been less studied. The present study aimed at investigating the time dependent changes in cognitive performance on prolonged stay at high altitude and its correlation with electroencephalogram (EEG) and plasma homocysteine. The study was conducted on 761 male volunteers of 25–35 years age who had never been to high altitude and baseline data pertaining to domain specific cognitive performance, EEG and homocysteine was acquired at altitude ≤240 m mean sea level (MSL). The volunteers were inducted to an altitude of 4200–4600 m MSL and longitudinal follow-ups were conducted at durations of 03, 12 and 18 months. Neuropsychological assessment was performed for mild cognitive impairment (MCI), attention, information processing rate, visuo-spatial cognition and executive functioning. Total homocysteine (tHcy), vitamin B_12_ and folic acid were estimated. Mini Mental State Examination (MMSE) showed temporal increase in the percentage prevalence of MCI from 8.17% on 03 months of stay at high altitude to 18.54% on 18 months of stay. Impairment in visuo-spatial executive, attention, delayed recall and procedural memory related cognitive domains were detected following prolonged stay in high altitude. Increase in alpha wave amplitude in the T3, T4 and C3 regions was observed during the follow-ups which was inversely correlated (r = −0.68) to MMSE scores. The tHcy increased proportionately with duration of stay at high altitude and was correlated with MCI. No change in vitamin B_12_ and folic acid was observed. Our findings suggest that cognitive impairment is progressively associated with duration of stay at high altitude and is correlated with elevated tHcy in the plasma. Moreover, progressive MCI at high altitude occurs despite acclimatization and is independent of vitamin B_12_ and folic acid.

## Introduction

With the constant rise in human population at high altitude due to strategic or military reasons, athletic training or call of duty, there is a necessity to investigate the effect of prolonged exposure to high altitude on cognitive and physiological health. Studies on effect of ascent to high altitude on mountaineers show decline in physical and mental performance even on short durations of exposure lasting from few days to <03 months that is greatly influenced by rate of ascent and duration of stay at high altitude [1]. Acute exposure to decreased partial pressure of oxygen at high altitude leads to a myriad of neuro-physiological and psychological derailments including special senses such as vision, higher functions such as memory and affective behavior such as mood [2], [3]. A number of studies have shown that verbal and visual short-term memory as well as recall is impaired on short duration of exposure to high altitude starting from 2500 m [4]. Moreover, there is burgeoning evidence on persistent defects in neuropsychological functions on return to sea level after brief periods of hypoxic exposure at high altitude [5]. However, considering the propositions on the onset of physiological acclimatization on prolonged stay at high altitude, the effect of chronic exposure to high altitude on cognitive performance remains to be studied.

Homocysteine, a product of methylation reactions involving transfer of methyl group from methionine in several metabolic pathways viz., DNA methylation and synthesis of carnitine, coenzyme Q10, creatine, choline, epinephrine etc., has currently emerged as an independent risk factor for stroke, neurodegeneration, vascular dementia, Schizophrenia and Alzheimer's disease [7]–[11]. Elevated circulating levels of total homocysteine (tHcy), has been associated with decline in cognitive performance in several geriatric population based studies [12]–[14]. Our previous studies have shown increase in total homocysteine (tHcy) on prolonged stay of 18 months at altitudes >4500 m [6]. However, association of tHcy in humans with cognitive performance during exposure to high altitude still remains to be investigated.

We, therefore, aimed at temporally assessing the domain specific changes in cognitive performance of sea level natives after induction and prolonged stay at high altitude. A longitudinal study was performed on a relatively large cohort of 761 Indian lowlander population who were inducted from 240 m MSL (Delhi) to an altitude of 4200–4500 m (Ladakh region) and stayed there for 18 months. A battery of psychological tests was administered at (240 m MSL) and during the follow-ups at 03, 12 and 18 months post induction to high altitude to assess prevalence of mild cognitive impairment (MCI) and domain specific changes in cognition. Electroencephalogram (EEG) recordings were performed to correlate the observed psychological traits with electrophysiological activity of different brain regions. tHcy, Vit B_12_ and folic acid levels were estimated at 250 m MSL during acquisition of baseline data and at 03, 12 and 18 months stay at high altitude to determine their co-relation with cognitive performance at high altitude.

## Methods

### Ethical clearance

The experimental design and procedures for conducting the experiment were approved by the ethics committee on human investigation of Defence Institute of High Altitude Research (DIHAR), Leh-Ladakh, India in compliance with Indian Council of Medical Research (ICMR) guidelines. Informed written consent was obtained from all the participants who volunteered for the study. The data acquired was shared with the volunteer and archived in the institute for future reference.

### Study population

**Figure 1:**
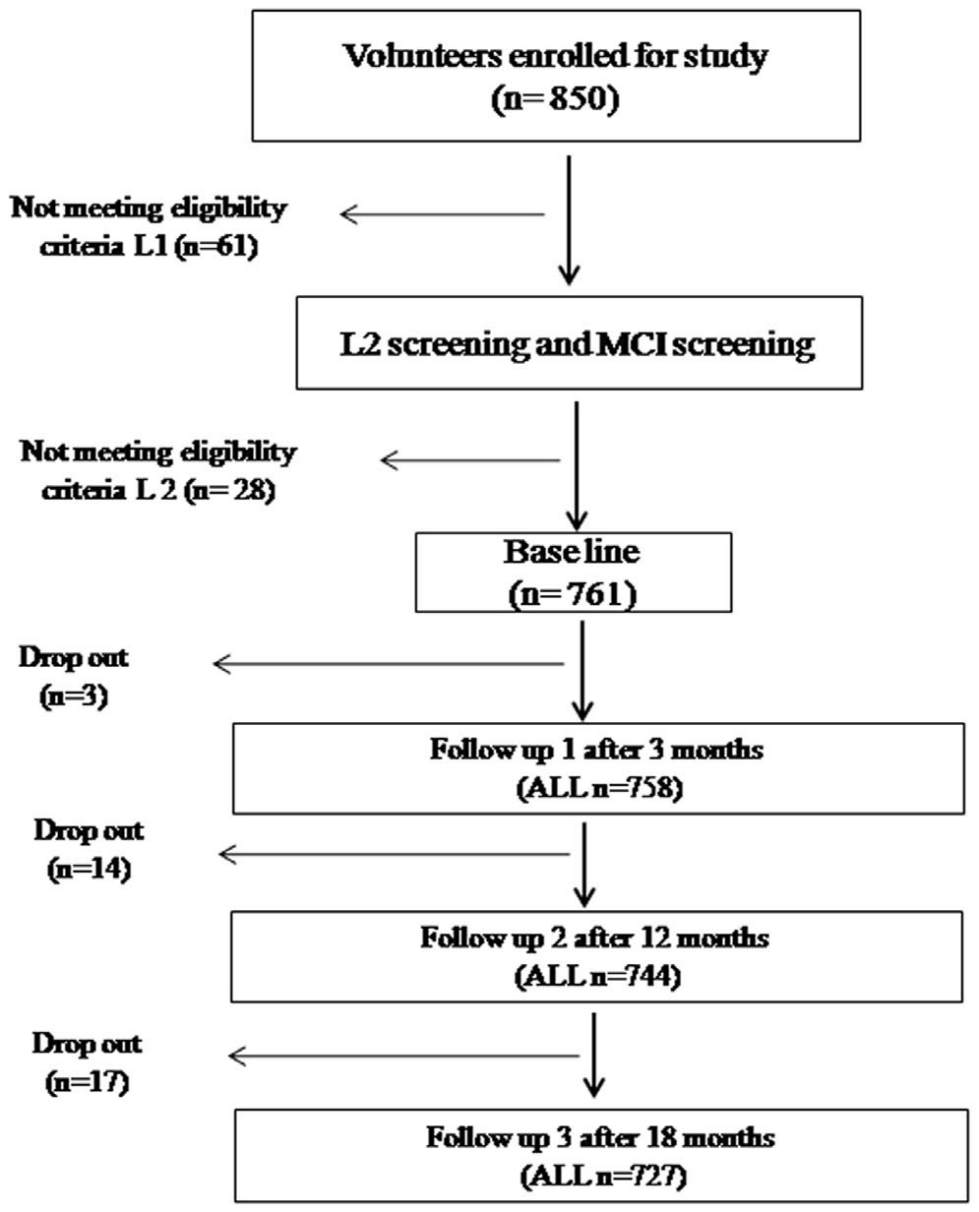
Flow chart showing study design and study population size at baseline and follow-ups.

This study was designed to conduct a longitudinal follow-up of a considerably large cohort to examine the temporal effect of stay at high altitude on the cognitive performance of healthy lowlander human population and its correlation with tHcy. The volunteers were individually informed about the purpose of the study, procedures to be administered and the expected outcome of the study and informed consent was obtained. 850 male participants initially enrolled for the study and baseline data was acquired at an altitude of 240 m MSL in Delhi and adjoining areas in the month of April 2009. Preliminary screening was performed based on eligibility criteria L1 comprising of age, gender, education, monthly income and medical examination. Physical examination was performed in the presence of a clinician and a medical questionnaire comprising questions related to occurrence of chronic diseases, physical and physiological ailments, heart problem, stroke, epilepsy, head injury, drug abuse, psychological disorders and general health status was administered to the participants (Table 1). The participants following L 1 screening, were screened for compliance to eligibility criteria L2 comprising of core behavioral measures (CBM) like core alcohol consumption (section A), core smoking behavior (section C), and core physical activity (section P) in accordance with WHO guidelines [15]. Beck Depression Inventory (BDI) was administered to investigate the presence of hitherto undetected depression that could influence cognitive performance and the information was verified from close acquaintance of the volunteer [16]. Lake Louise Score for acute mountain sickness (AMS) was administered to the participants on arrival and during 07 days acclimatization at high altitude to negate possible occurrence of AMS symptoms [17]. Mini Mental State Examination (MMSE) was administered to the participants during acquisition of baseline data and individuals having MMSE score <25 were excluded from the study (Table 2). Liver function test viz., serum glutamic oxaloacetic transaminase (SGOT), serum glutamic pyruvic transaminase (SGPT); kidney function test viz., blood urea nitrogen and creatinine, lipid profiling and blood glucose test were performed according to standard procedures to rule out possible influence of these parameters on the cognitive performance during baseline data acquisition (Table 2). Of the 850 volunteers who enrolled for the study, baseline data was acquired from 761 volunteers who complied to the inclusion criteria viz., between the age group of 25–35 years with no history of medical complications and neurological disorders, drug abuse, stroke, epileptic seizures, brain injury or cognitive impairment and with 10–15 years of education (Figure 1). Volunteers were inducted to high altitude in the month of May 2009. Active follow-ups were conducted at 03 months (follow-up 1, August 2009), 12 months (follow-up 2, May 2010) and 18 months (follow-up 3, November 2010) of induction to high altitude regions of Ladakh. Follow-up studies after induction to high altitude were carried out in make shift set ups in the subject locations at altitudes ranging from 4200 m–4600 m above MSL, during May 2009 to November 2010, that included remote areas in Ladakh. Similar ambience was maintained during each phase of the study to minimize influence of external environment on the cognitive test battery. Female subjects were not recruited to rule out the influence of estrogen and other hormonal factors on the results of the study.

**Table 1:** Basic inclusion criteria (Eligibility criteria L1) for volunteers enrolled for the study.

Parameters	Value
Age (Years)	27.3±2.34
Birth altitude range (Min-Max, M, MSL)	213–305
Duration of stay at high altitude (Years)	0
Education (Years)	12±2
Income per month (INR)	18000±2243
**Medical History/Health Status**	
Any serious health illness	No
Head injury resulting in loss of consciousness	No
Any form of seizers, delirium tremens or convulsions	No
Allergies to medication, foods, animals, chemicals or other agents	No
Lung diseases such as asthma, emphysema, or chronic bronchitis	No
Surgeries or hospitalizations	No
Hypertensions	No
Diabetes	No
Viral hepatitis	No
Dementia/Memory Impairment	No
Stroke/Infarction/Cerebral Hemorrhage	No
Kidney disease	No
GERD symptoms	No
Chest pain	No
Congenital heart disease	No
Neurological Problem/Epilepsy	No
Cancer	No
Heart attack or any heart problem	No
Familial disorders	No

**Table 2:** Eligibility Criteria L2 comprising of core behavioral measures (CBM), Beck Depression Inventory (BDI), Mini Mental State Examination (MMSE), Lake Louise Score for Acute Mountain Sickness and baseline characteristics for kidney function, lipid profile, liver function and blood glucose.

Core Behavioral Measures (%)	Values
*Core Alcohol Consumption (Section A)*	
No Consumption (%)	97.7
Mild Consumption (%)	2.3
Moderate Consumption (%)	Nil
Severe Consumption (%)	Nil
*Core Tobacco Use (Section C)*	
No Smoker	95.7
Mild Smoker (%)	4.3
Moderate Smoker (%)	Nil
*Core Physical Activity(Section P)*	
Mild (%)	82.43
Moderate (%)	16.52
Severe (%)	1.05
Severe Smoker (%)	Nil
Beck Depression Inventory (BDI)(Score)	5.23±1.34
Mini Mental State Examination(Score)	28.01 ±0.72
Lake Louise Score	0
**Biochemical Estimations**	
*Kidney Function Test*	
Blood Urea Nitrogen (6–20 mg/dl)	9.3±1.1
Creatinine (0.9–1.3 mg/dl)	1.01±0.11
*Lipid Profile*	
Cholesterol (<200 mg/dl)	139.9±24.1
Triglycerides (<150 mg/dl)	82.3±26.2
HDL (40–60 mg/dl)	46.5±5.3
LDL (<100 mg/dl)	81.4±12.3
Chol/HDL Ratio (<3.3)	2.95±0.28
LDL/HDL Ratio (<3.0)	1.88±0.5
VLDL (≤30 mg/dl)	16.5±5.2
*Liver Function test*	
SGOT (15–37 U/L)	27.8±5.0
SGPT (30–65 U/L)	40.16±7.57
*Blood Glucose*	
Fasting Glucose	83.02±3.91

Values depicted either as percentage or as Mean±SD of the study population.

### Physiological measurements

The participants were made to relax in sitting position 05 minutes prior to acquisition of physiological data. Systolic and diastolic blood pressure (SBP and DBP), pulse rate and oxygen saturation of hemoglobin (SpO_2_) were recorded in sitting position. Height and weight were measured using portable anthropometer and digital weighing machine respectively. The body mass index (BMI) was then calculated and expressed in kg/m^2^.

### Neuropsychological assessment

MCI was diagnosed following multidisciplinary meetings involving neuropsychologists, researchers and clinicians based on a battery of neuropsychological tests and physical examination. Neuropsychological battery was administered by trained professionals and analysis of data was performed independently by a statistician.

#### MCI Screening Tests

Subjects were screened with Mini mental State Examination (MMSE) which is a brief 30-point questionnaire for assessment of MCI [18]. Subjects obtaining score greater than and equal to 25 out of 30 were considered to be cognitively normal while subjects scoring below 25 were considered to be MCI. A recently designed and validated 45-point Multi Domain Cognitive Screening Test (MDCST) was also administered simultaneously to assess nine different cognitive domains (5 point for each domain) viz., orientation, memory, visuo-spatial executive, object recognition, attention, delayed recall, language, coordination and procedural memory [19]. Subjects scoring 36 and above out of 45 point scale were considered as cognitively normal participants (CNP) while scores below 36 were considered as MCI.

#### Raven Standard Progressive Matrices (RSPM)

RSPM was administered to assess general intelligence and visuo-spatial cognition according to the procedure suggested by Raven et al [20]. Completion of the test queries required involvement of working memory, category shift, mental flexibility, problem solving, abstraction and reasoning. The test comprised of five sets (A to E) of 12 items each (e.g. A1 through A12), with items within a set becoming increasingly difficult and requiring greater cognitive abilities to encode and analyze information. Scoring was based on duration of completion and total number of correct attempts. All items were presented in black ink on a white background.

#### Clock Drawing Test (CDT)

Visuo-spatial ability which is primarily attributed to the functioning of the parietal lobe was measured by using CDT as suggested by Schulman [21]. In brief, the participants were asked to fill in the numbers and then set the time to ‘08:20’ in a pre-drawn circle (approximately 10 cm in diameter). A score of ‘0’ implied an intact clock while score of ‘1’ and above implied cognitive impairment. Errors like omissions of clock hands, perseverations, rotations, misplacement of digits, distortions, substitutions and additions were also considered during the scoring.

#### Bender Visual Motor Gestalt Test (BGT)

BGT was administered to determine visual motor maturity and motor skills [22], [23]. The test consisted of nine figures on a 3×5 dimensioned card. Each figure was shown to the subjects who were asked to copy them onto a piece of blank paper and scoring was done using Hain's method. Errors were counted on the basis of 15 different signs like perseveration, rotation, concretism, added angles, separation of lines, overlap, embellishment, partial rotation, omission, abbreviation, separation, absence or erasure, closure and point of contact. Each sign was scored only once and the final score was calculated. The total error score ranged from 0–34.

#### Serial Digit Learning Test (SDLT)

SDLT comprised of a 9-digit series presented orally to the participants at the rate of 1 digit per second, and the participants were asked to learn and repeat them in their given order [24]. The procedure was terminated if the participant repeated the series correctly in two consecutive trials or when the participant could not repeat the series in 12 consecutive trials. The performance on SDLT required utilization of mnemonic strategies and this test therefore, served as a tool for assessing attention, learning and memory.

#### Stroop Color Word Interference Test-TBAG Form (SCWT)

SCWT was administered according to the procedure suggested by MacLeod to assess the information processing rate, parallel processing of attended and unattended stimuli and attention [25]. There was no time limit to complete a subtask. The time needed to complete each Stroop subtask served as dependent measures (Stroop I, Stroop II, and Stroop III respectively). The examiners did not point out errors made during the test and participants spontaneously corrected themselves if they noticed an error. The number of errors that were not self corrected was also recorded for each Stroop subtask (Error I, Error II and Error III respectively). Poor performance was marked by higher inference score in this test.

#### Trail Making Test (TMT)

TMT was administered to assess visual search speed, scanning, speed of processing, mental flexibility, attention as well as executive functioning. TMT was bifurcated into two parts: TMT-A required an individual to draw lines sequentially connecting 25 encircled numbers distributed in ascending sequential order on a sheet of paper until he reached the circle labeled ‘END’. TMT-B comprised of circled alphabets ranging from A to L and 1–13 and the participant was asked to alternate between numbers and letters (e.g., 1, A, 2, B, 3, C, etc.) in ascending sequential order until he reached the circle labeled ‘END’. The time required to complete the task was considered as the test score [26].

#### Verbal Fluency Test (VFT)

VFT was performed to evaluate the executive function of the frontal and temporal lobe. Participants were asked to say as many words as possible from a category (e.g., birds, animals etc.) in a given time (60 seconds). Higher value of score in this test was dependent on the number of words named by the subject in the given time [27].

### EEG data acquisition and analysis

Electrodes were placed according to the universal 10–20 system and the electroencephalogram (EEG) signals were recorded from electrodes placed on scalp positions viz. frontal (Fp1, Fp2), central (C3, C4), temporal (T3, T4) and occipital (O1 and O2) sites using Neurotravel EEG System (ATES medica Device, Verona, Italy). Subjects were made to relax in sitting position for five minutes following which EEG signal was recorded with closed eyes for ten minutes. The EEG was recorded with a 0–30 Hz bandpass and digitized using a 500 Hz sampling rate. EOG and EMG signals were also recorded to remove artifacts due to eye and muscle movements during signal processing. The algorithm for digital signal processing used a 1024 point Fast Fourier Transform with Hamming windows (−53 dB stop band, filter degree 1068, transition bandwidth 0.622 Hz), in 30 s block. Amplitude analysis was done for all the frequency bands employing Neurotravel map software (V2.5.2.26, ATES medica device, Verona, Italy) after eliminating different motion artifacts. Continuous EEG raw signal was divided into epochs of 30 seconds and independent component analysis was applied to remove eye blinks and muscle artifacts.

### Biochemical estimations

Blood was collected from the volunteers in aseptic conditions by venous puncture after an overnight fast. The tHcy levels in plasma was determined using high pressure liquid chromatography (HPLC) (Waters, Milford, MS, USA) according to the procedure suggested by Refsum et al. [28]. System detectors were adjusted for excitation of 385 nm and an emission of 515 nm to capture fluorescence separated compounds. tHcy was estimated from a calibration curve obtained from known amounts of homocysteine [28]. Serum was extracted from blood samples by centrifugation at 6000 rpm for 5 minutes. Serum levels of folic acid and vitamin B_12_ were determined by a radio assay kit (Bio-Rad, Richmond, CA), according to the manufacturer's instructions. The concentration of vitamin B_12_ and folic acid in serum was determined by extrapolation from the standard curve.

### Statistical analysis

The participants were categorized into two separate sub groups viz. CNP and MCI based on their psychological scores with respect to globally accepted normal values. Descriptive analysis was done for the psychological scores. Mean, standard deviation and standard error of mean was calculated for each group. Statistical analysis was performed using ANOVA with Duncan's Post Hoc test for comparisons between groups using SPSS 17.0 Statistic software (SPSS, Chicago, IL, USA). Relations between variables were analyzed by calculating the Pearson product- moment correlation coefficients. *P*-values <0.05 (two tailed) were considered to be significant. The data was archived in the central records keeping centre of DIHAR.

## Results

### Physiological characteristics of the study population

Physiological tests (Table 3) were performed at baseline before induction and during all the follow-ups. Simple t-test was done between baseline and follow-up recordings independently. Oxygen saturation was found to be low during all the follow-ups conducted at high altitude (*P*<0.05) when compared to baseline values. Systolic and diastolic blood pressure was within physiological range, though a significant change in pulse rate was observed during the follow-ups at high altitude. No change in waist/hip ratio and BMI was observed during the study period.

**Table 3:** Physiological Characteristics at baseline and during different durations of stay at high altitude.

Parameters	Baseline	Follow-up 1 (After 3 months)	Follow-up 2 (After 12 months)	Follow-up 3 (After 18 months)
SpO_2_ (%)	97.40±0.62	89.03*±0.51	91.24*±0.39	91.31*±0.59
PULSE RATE	77.18±1.54	80.64*±0.97	80.60*±1.87	82.25*±2.29
W/H Ratio	0.90±0.04	0.89±0.03	0.88±0.06	0.89±0.08
BMI	23.13±1.30	23.46±1.61	21.86±1.37	21.13±1.14
SBP (mm Hg)	120±1.23	122±1.83	122±1.75	121±1.77
DBP (mm Hg)	81±1.01	83±2.18	82±1.60	82±1.13

Values depict Mean±SEM,

*denotes *P*<0.05 when compared to baseline values.

### Neuropsychological changes on prolonged stay at high altitude

**Figure 2:**
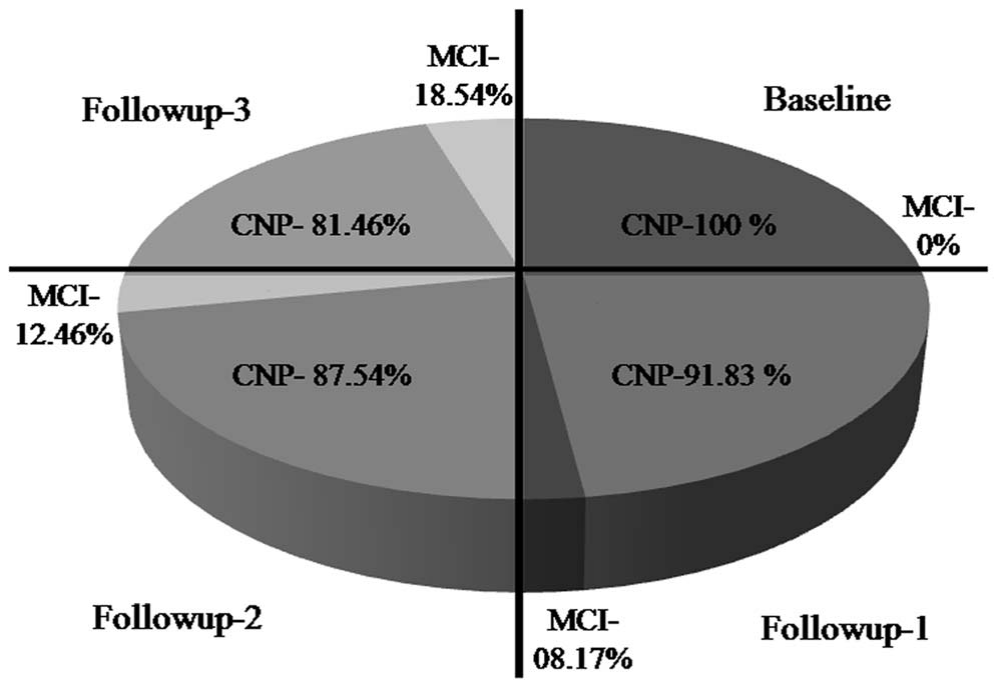
Percentage prevalence of mild cognitive impairment in study population during baseline and follow-ups. Percentage of participants with mild cognitive impairment (MCI) increased progressively with duration of stay at high altitude.

Table 4 depicts the mean MMSE and MDCST scores of the study population at baseline and during the follow-ups. The results showed significant decrease in the mean MMSE score indicating increase in MCI during second (24.2±0.84, *P*<0.05) and third (24.4±0.45, *P*<0.05) follow-up when compared with the baseline scores (28.2±0.27). As indicated in figure 2, there was a persistent temporal increase in the percentage prevalence of MCI that ranged from 8.17% in the first follow-up, to 12.46% in the second follow-up and further increasing up to 18.54% during the third follow-up as determined by MMSE (figure 2). The visuo-spatial executive, attention and problem solving domains were most affected during stay at high altitude and the decline was progressive as shown in Table 4. Considerable decline in delayed recall and procedural memory functions was also observed during the second (*P*<0.05) and third follow-ups (*P*<0.05). Derailment in orientation was observed only during the third follow-up when compared with baseline CNP. Individuals showing cognitive decline in any of the affected domains did not revert back to normal during the period of investigation. One-way ANOVA of the data of RSPM test revealed a significant difference in response values between the baseline (41.03±7.65) and the follow-ups (65.01±10.03 for follow-up 1, 67±9.3 for follow-up 2, and 69.81±6.93 for follow-up 3, *P*<0.05) as shown in Table 4. One-way ANOVA with repeated measures also detected a significant difference in CDT values of the baseline and the follow-ups (*P*<0.05). An intact clock carrying score ‘0’ was obtained by the CNPs during follow-ups. Significant increase in CDT scores of MCI was observed during first (1.93±0.39, *P<0.05*) and second (2.17±0.21, *P*<0.05) follow-up due to the errors like misplacement of digits, distortions, substitutions and additions. Errors like omissions of clock hands, perseverations, and rotations contributed to increase in CDT scores during third follow-up (2.30±0.51, *P*<0.05).

**Table 4:** Assessment of cognitive impairment using Mini Mental State Examination (MMSE), Multi Domain Cognitive Screening Test (MDCST), Raven Standard Progressive Matrices (RSPM), Clock Drawing Test (CDT), Bender Visual Motor Gestalt Test (BGT), Facial Recognition Test (FRT), Serial Digit Learning test (SDLT), Stroop Color Word Interference Test-TBAG Form (SCWT), Verbal fluency test (VFT), Visual-aural digit span test-B (VADS-B) during baseline and follow-ups.

PARAMETERS	Baseline (n = 761)	Follow up 1 (n = 758)		Follow up 2 (n = 744)		Follow up 3 (n = 727)	
		CNP (n = 696)	MCI (n = 62)	CNP (n = 651)	MCI (n = 93)	CNP (n = 592)	MCI (n = 135)
MMSE	27.20±0.27	27.20±0.43	25.20±0.62	28.40±0.92	24.20*±0.84	28.00±0.52	24.40*±0.45
MDCST	38.63±2.87	37.50±2.78	32.63±1.73	37.43±2.04	30.70*±2.87	38.37±1.82	28.01*±3.07
I. Orientation	4.50±0.49	3.88±0.86	4.13±0.00	4.26±0.69	4.10±0.88	4.55±0.51	3.19*±0.81
II. Visuospatial Executive	3.98±0.97	3.13±1.55	2.13*±0.41	3.78±0.90	2.20*±0.48	3.79±0.90	2.00*±0.25
III. Attention & problem solving	4.53±0.88	5.00±0.00	3.47*±0.28	4.57±0.79	3.40*±0.39	4.41±0.56	3.16*±0.27
IV. Memory Registration & immediate recall	4.78±0.48	5.00±0.00	4.54±0.28	4.87±0.34	4.80±0.42	5.00±0.00	4.58±0.42
V. Object recognition & remote memory	4.83±0.38	4.88±0.35	5.00±0.00	4.83±0.39	4.80±0.42	4.83±0.38	4.95±0.23
VI. Mind Body Coordination and Learning	3.85±1.42	3.75±0.89	3.33±0.58	3.91±0.85	2.60*±0.97	3.24±0.79	1.95*±1.08
VII. Language	3.60±1.15	3.50±1.31	2.74±1.00	3.43±0.95	2.90±1.45	3.69±1.14	2.95±1.22
VIII. Delayed Recall	4.70±0.30	4.38±0.92	4.00±1.00	4.00±0.95	3.40*±0.37	4.21±0.94	3.13*±0.30
IX. Procedural Memory	4.88±0.22	4.86±0.24	4.64±0.24	4.78±0.28	3.50*±0.44	4.59±0.32	2.89*±0.54
VISUO SPATIAL ABILITY							
RSPM	41.03±7.65	38.21±8.21	65.01*±10.03	43.84±9.13	67*±9.30	40.61±5.72	69.81*±6.93
BGT	1.21±0.92	1.52±1.09	1.87±1.01	1.08±0.79	1.04±0.74	1.92±1.27	1.51±1.07
CDT	0	0	1.93*±0.39	0	2.17*±0.21	0	2.30*±0.51
ATTENTION							
SDLT	14.83±4.12	12.97±3.47	5.19*±3.12	14.38±3.17	6.53*±4.07	15.03±4.21	5.07*±3.81
SCWT	32.83±11.07	31.40±9.25	69.41*±9.72	33.71±10.86	73.19*±12.71	30.51±11.09	71.28*±14.26
Trail making Test PART A	22.13±6.03	20.82±3.52	23.21±7.47	24.91±8.34	28.49±7.31	21.37±6.57	20.67*±6.39
Trail making Test PART B	43.71±8.21	39.91±5.97	41.52±6.39	40.82±5.70	38.64±7.48	40.36±6.31	39.72±6.92
MEMORY AND LANGUAGE ABILITY							
VFT-Birds	17.56±2.81	18.31±5.72	17.28±3.18	17.53±4.72	18.94±3.85	18.71±5.03	17.27±2.93
VFT-Animals	28.51±3.62	27.04±4.29	25.93±3.75	28.39±4.76	27.21±2.21	25.01±7.03	26.54±3.73

Value depicts Mean±SD,

*denotes *P*-value <0.05 when compared to baseline data.

Consecutive trial was taken as the variable in the SDLT, a measure of attention, where the scores were found to decrease significantly in MCI group during the follow-ups (5.19±3.12, for follow-up 1, 6.53±4.07, for follow-up 2, and 5.07±3.81, for follow-up 3, *P<0.05*). No difference in scores of learn and repeat task of SDLT was observed between the baseline and CNP during follow-ups (14.83±4.12, for baseline, 12.97±3.47, for follow-up 1, 14.38±3.17, for follow-up 2, and 15.03±4.21, for follow-up 3, *P>0.05*). However, response duration in Stroop III of SCWT increased in MCI during the follow-ups. One-way ANOVA revealed significant delay in response between baseline and MCI during follow-ups in the Stroop III (69.41±9.72, for follow-up 1, 73.19±12.71, for follow-up 2, and 71.28±14.26, for follow-up 3, *P*<0.05). A difference in language ability over the entire reading and learning sequences was observed only at third follow-ups (0.98±0.73, *P*<0.05). No significant change in scores of MCI was observed during first (2.47±0.79, *P*>0.05) and second (2.79±0.81, *P*>0.05) follow-up due to less errors.

### Electrophysiological changes at high altitude

**Figure 3:**
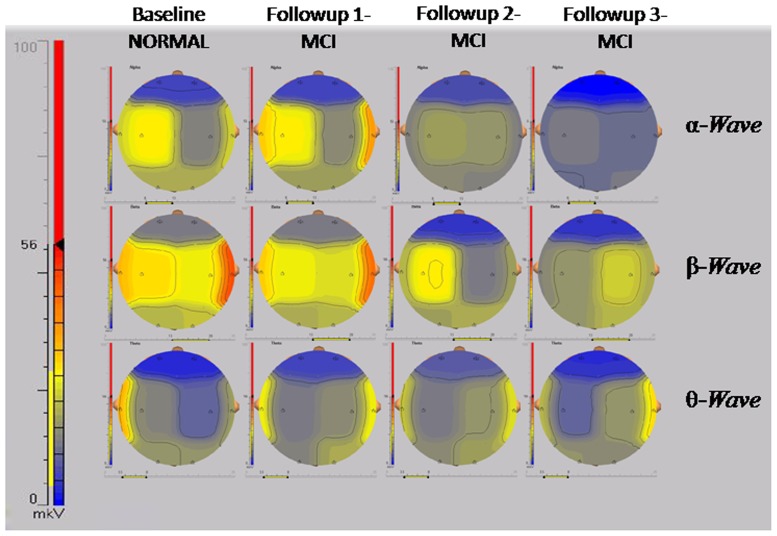
Representative brain maps of alpha, beta and delta components of EEG of the same participant during baseline and follow-ups. Scale bar shows regional activity in mkV.

Analysis of EEG data acquired from the participants in eyes closed position for theta wave amplitude showed decrease in the T3 region (95% CI 9.82–13.44) but a corresponding increase in the T4 region (95% CI 17.95–21.49) following 3 months of induction to altitude. On the contrary, there was an increase in alpha wave amplitude in the T3 (95% CI 17.43–19.63), T4 (95% CI 18.83–21.2) and C3 (95% CI 20.78–22.46) regions. In addition, a strong inverse correlation (r = −0.68) was observed between alpha wave amplitude and both MMSE and MDCST scores. In addition to the decreased theta wave amplitude in the T3 region (95% CI 9.44–11.82) and increased amplitude in T4 region (95% CI 21.52–22.84) and increased alpha wave amplitude in the T3 (95% CI 20.93–22.84), T4 (95% CI 20.30–22.44) and C3 (95% CI 21.87–23.95) region, we also observed an increase in alpha wave amplitude in the C4 region (95% CI 22.03–25.65) along with decrement in beta wave amplitude in the T3 (95% CI 9.44–11.82) and T4 (95% CI 9.44–11.82) regions (Table 5). Interestingly, on prolonged stay at high altitude for 18 months, no change in theta and beta wave amplitude was observed as shown in figure 3. The alpha wave amplitude however continued to remain high in all the regions of interest when compared to the baseline values as shown in (Table 5).

**Table 5:** Amplitudes (µV) of EEG wave components at different electrode positions.

ROI	Wave	Baseline		Followup 1		Followup 2		Followup 3	
		Normal	MCI	Normal	MCI	Normal	MCI	Normal	MCI
**T3**	**Delta**	41.44±1.61	NA	41.28±1.88	39.63±2.05	40.12±0.51	41.59±1.71	41.13±1.35	39.31±0.41
	**Theta**	14.23±2.23	NA	15.14±1.33	11.63±1.81	14.11±0.75	10.63±1.19	14.31±1.07	13.6±1.72
	**Alpha**	15.11±1.73	NA	16.21±1.02	18.53*±1.1	14.23±1.92	21.91*±0.93	15.71±1.91	24.79*±1.8
	**Beta**	6.62±1.84	NA	7.78±1.12	6.94±0.58	6.18±1.92	4.09*±0.58	6.41±0.4	5.93±1.12
**T4**	**Delta**	45.8±1.43	NA	41.15±1.31	40.13±0.39	38.12±1.91	40.18±0.49	41.06±1.05	39.01±1.01
	**Theta**	14.68±1.00	NA	15.35±1.32	19.72±1.77	15.41±1.41	21.19±1.62	15.98±1.51	16.65±2.67
	**Alpha**	14.56±1.97	NA	16.16±1.37	20.02*±1.19	15.01±1.3	21.37*±1.07	15.22±0.93	23.14*±1.33
	**Beta**	6.45±1.89	NA	6.2±1.5	6.4±1.13	6±0.9	4.4*±0.23	5.5±1.92	5.01±0.92
**C3**	**Delta**	39.4±1.74	NA	39±1.23	38.1±0.92	39.14±1.21	39.06±0.90	36.09±1.67	37.08±1.07
	**Theta**	14.17±1.04	NA	14.99±1.47	13.89±1.92	15.16±1.7	14.09±1.01	14.01±0.75	14.89±1.57
	**Alpha**	16.63±1.64	NA	15.14±1.08	21.62*±0.84	15.21±0.68	22.91*±1.04	16.23±1.09	21.03*±1.00
	**Beta**	6.7±1.62	NA	6.3±1.11	6.00±1.92	6.03±1.39	5.9±0.93	6.89±1.08	5.08±1.33
**C4**	**Delta**	39.33±1.05	NA	38.13±2.01	40.61±1.41	39.49±1.24	39.73±1.01	41.09±1.17	38.53±1.25
	**Theta**	13.19±1.73	NA	12.71±1.59	11.93±2.84	13.11±1.04	12.19±1.21	13.74±1.42	12.04±1.09
	**Alpha**	16.34±1.96	NA	16.05±1.03	19.91±2.42	15.35±1.03	23.59*±1.81	14.76±1.23	21.06*±1.19
	**Beta**	6.2±1.24	NA	6.7±1.38	6.18±1.39	6.79±1.34	6.58±0.99	5.17±1.79	6.17±1.82

Value depicts mean ± SEM,

*denotes *P*-value <0.001 when compared to baseline values. ROI: region of interest.

### Association of biochemical parameters with MCI

**Figure 4:**
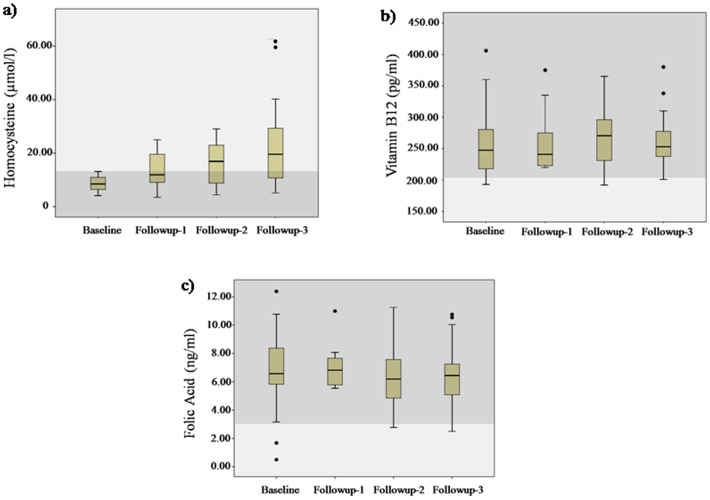
Population distribution of a) Homocysteine b) Vitamin B_12_ and c) Folic acid during baseline and follow-ups. * denotes *P*-value <0.001 when compared to baseline data.

The box plots in figure 4 depict the biochemical profile of tHcy, vitamin B_12_ and folic acid showing progressive increase in the prevalence of hyperhomocysteinemia in the study population. The results showed elevated mean tHcy levels in all the follow-ups (13.54±2.46, *P<0.05* for first follow-up, 16.29±5.42, *P*<0.05 for second follow-up and 21.42±3.74, *P*<0.001 for third follow-up), when compared with baseline CNP (8.64±0.76). No significant change was observed in the serum vitamin B_12_ and folic acid levels during the follow-ups (*P*>0.05). Table 6 illustrates Pearson's correlation between MMSE score and plasma tHcy, serum vitamin B_12_ and serum folic acid. tHcy levels in all the three follow-ups showed a progressive positive association with MCI when compared to the baseline CNP. Plasma levels of tHcy were inversely correlated with MMSE score in all the follow-ups (r = −0.431; *P*<0.001 for first follow-up, r = −0.345; *P*<0.05 for second follow-up, and r = −0.838; *P*<0.001 for third follow-up). No significant correlation was found between MMSE score and vitamin B_12_ or folic acid.

**Table 6:** Correlation of homocysteine, vitamin B_12_ and folic acid with mild cognitive impairment.

	MMSE Score	
	*Correlation*	
Follow-ups	Correlation Coefficient (r)	*P*-value
*HOMOCYSTEINE*		
Baseline		
First	−0.431	**0.002** *
Second	−0.345	**0.003** *
Third	−0.838	**0.001** *
*VIT B_12_*		
Baseline		
First	0.098	0.875
Second	0.117	0.852
Third	0.21	0.472
*FOLIC ACID*		
Baseline		
First	0.532	0.356
Second	0.108	0.737
Third	0.105	0.721

*denotes *P*-value <0.05 when compared to baseline data. ‘r’ denotes Pearson product moment correlation analysis with no adjustment.

## Discussion

High altitude is characterized by a decrease in partial pressure of oxygen leading to reduced oxygen saturation of blood and stunted oxygen availability to the tissues resulting in a physiological condition called hypobaric hypoxia [1], [3], [6]. The brain, in particular, is known to be most susceptible to oxygen deficiency. Despite the fact that a considerable size of low lander human population stay at high altitude for long durations due to occupational requirements, the effect of hypobaric hypoxia on cognition of these individuals has not been adequately understood. In this prospective study of 761 healthy non-demented low lander population with normal MMSE and MDCST scores who were inducted to high altitude environment of 4200–4600 m for 18 months due to occupational requirements, there was an irreversible duration dependent increase in prevalence of MCI that was correlated with duration of stay at high altitude. Assessment of cognitive domains using Mini mental State Examination and neuropsychological battery comprising of Multi Domain Cognitive Screening Test, Raven Standard Progressive Matrices, Clock Drawing Test, Bender Visual Motor Gestalt Test, Serial Digit Learning test, Stroop Color Word Interference Test-TBAG Form, Trail Making Test and Verbal Fluency Test showed impairment in visuo-spatial executive, attention, delayed recall and procedural memory related cognitive domains.

Previous reports on effect of hypobaric hypoxia on human physiology suggest occurrence of acclimatization after 3–5 days of induction to high altitude. Onset of acclimatization is considered to be an outcome of physiological adjustments to the hypobaric conditions at high altitude for evading the deleterious effects of oxygen deficiency [29]. Our findings, however, reveal that despite physiological acclimatization, prolonged stay at high altitude results in derailment of cognitive performance in sea level natives. The domain specific assessment of prospective cognitive decline at high altitude revealed early onset of deficits in visuo-spatial executive and attention while decline in cognitive domains related to delayed recall and procedural memory were observed only after 12 months of stay at high altitude. Assessment by RSPM showed a derailment in executive functioning on prolonged stay while decrease in scores of CDT test indicates derailment of constructional praxis, indicating altered function of parietal lobe. Increase in SDLT and decrease in SCWT scores following stay at high altitude indicated towards alteration in medial temporal lobe, hippocampus and frontal lobe activity resulting in decreased attention. Though no detectable change was observed in the memory domain on 03 months and 12 months of stay at high altitude as assessed by verbal fluency test, decline in comprehension was observed following 18 months stay indicating that long term memory impairment onsets on longer durations of stay at high altitude. These findings are in agreement with previous reports by Zhang et al (2011) reporting impairment in immediate recall following stay for 06 months at an altitude of 2260 m [30]. However, contrary to the findings on minimal effect of high altitude on long term memory, we observed a decline in language and comprehension on prolonged stay of 18 months at 4200 m. This discrepancy may be attributed to the higher altitude and longer duration of stay in hypobaric conditions during the present study.

Contradictory to reports on restoration of cognitive performance following acclimatization, the impairment in the cognitive domains was irreversible during the entire period of our study [31]. Our results, however, find support from reports of “Everest-Comex 97” during which climbers participating in a simulated climb from sea level to 8,848 m over a 31 day period showed selective impairment of mental learning processes related to cortico-limbic system rather than basal ganglia-sensorimotor system function [32]. Similar attentional changes in a group of volunteers staying at an altitude of 6,542 m for 21 days has also been previously reported [5]. Reports on MRI scans also showed abnormalities in several brain areas and occurrence of cortical atrophy following ascent to high altitude [33], [34].

In addition to assessing the psychological changes associated with cognition, we also attempted to determine the electrophysiological correlates for the observed psychological changes. This is probably the first report on increase in alpha wave amplitude on prolonged stay of acclimatized low landers at high altitude for 18 months that in turn is positively correlated with MCI. Our study finds support from previous reports that show increase in P300 latency suggesting that hypoxia causes slowing of the signal processing at 4300 m, and magnitudes of the effects are altitude dependent [35]. The P300 (P3) wave is an event related potential component elicited in the process of decision making and is linked to a person's reaction time in response to stimuli. However, most of the studies on effect of hypobaric hypoxia on electrophysiological activity of the brain have been conducted on small cohorts with the duration of stay ranging from a few days to 3 months.

The present study also provides evidence for elevation in tHcy which was positively correlated to MCI and duration of stay at high altitude. Consistent to our previous report, the tHcy levels at high altitude were independent of serum vitamin B_12_ and folic acid levels which were in the normal range in all the participants of the study [6]. The optimal concentration of serum vitamin B_12_ and folic acid in the study population during the follow-ups at high altitude could be attributed to the administration of healthy diet recommended at high altitude. Though several studies have focused on hematological and metabolic effects of hypoxic exposure, no study has been conducted on the correlation of cognitive performance at high altitude with tHcy [36], [37]. However, increase in circulatory homocysteine has been previously reported in chronic stress models and age related neurological disorders [8]. The positive correlation between tHcy and MCI scores that was observed during the present study finds support from reports on association of hyperhomocysteinemia with cognitive decline in several neurodegenerative disorders including Multiple Sclerosis, Parkinson's disease and Alzheimer's disease [38]. Higher white matter lesion in atherosclerotic disease has also been attributed to hyperhomocysteinemia [39]. Considering previous reports on reduced white matter in motor function areas of the brain and occurrence of sagittal sinus thrombosis following ascent to high altitude, elevated homocysteine at high altitude could certainly play a key role in the psychological and pathophysiological manifestations of low oxygen in the brain functions [40], [41].

The present study, to the best of our knowledge, is the first report on association of hyperhomocysteinemia with decrease in cognitive performance of acclimatized sea level natives on prolonged stay for 18 months at high altitude. Besides that, considering the role of homocysteine in cardio-vascular as well as aging associated neurological disorders, our findings on occurrence of hyperhomocysteinemia on longer duration of stay at high altitude could be of considerable clinical relevance for occupational health at high altitude.

Homocysteine is metabolized either through the re-methylation pathway, which regenerates methionine through a folic acid mediated mechanism or the trans-sulfuration pathway that degrades homocysteine into cysteine and requires vitamin B_6_ [7]. Cysteine in turn is a precursor for synthesis of reduced glutathione (GSH) which is a major cellular antioxidant. Our previous studies have shown increase in oxidative stress and depletion in GSH following exposure to high altitude [42]. Since the increase in tHcy during the present study was independent of vitamin B_12_ and folic acid concentrations and increase in tHcy could have both protective effect by augmenting the antioxidant status as well as deleterious effect by mediating neurodegeneration, further investigations on metabolic pathways leading to increased homocysteine synthesis or reduced clearance may provide an insight into the biochemical changes occurring at high altitude. A major strength of the present study lies in the association of hyperhomocysteinemia with cognitive decline at high altitude which could be used for clinical diagnosis of MCI and provide valuable information on the health of the brain at high altitude. The findings of the present study also contradict the popular notion of acclimatization by revealing the duration dependent decrease in cognitive impairment at extreme altitudes, thereby necessitating investigations to understand the effect of chronic hypobaric hypoxia on the brain functions and metabolism.

## Conclusions

The findings of the present study indicate towards progressive mild cognitive impairment in acclimatized lowlanders on prolonged stay at high altitude. The mild cognitive impairment observed at high altitude is associated with increase in alpha wave amplitude and is correlated with hyperhomocysteinemia. These findings could have considerable clinical significance in diagnosis of mild cognitive impairment and assessment of cognitive health in high altitude conditions.
